# Reward‐specific learning parameters change across normative adolescent development and are blunted in youth with high risk for depression

**DOI:** 10.1111/jcpp.70086

**Published:** 2025-12-22

**Authors:** Holly Sullivan‐Toole, Jeremy M. Haynes, Helen Schmidt, Bart Larsen, Nathaniel Haines, Thomas M. Olino

**Affiliations:** ^1^ Psychology and Neuroscience Department Temple University Philadelphia PA USA; ^2^ Masonic Institute for the Developing Brain, Department of Pediatrics University of Minnesota Minneapolis MN USA; ^3^ Department of Psychology Georgia Southern University Statesboro GA USA; ^4^ Bayesian Beginnings LLC Columbus OH USA

**Keywords:** reward learning, reinforcement learning, adolescence, learning rate, maternal depression, Iowa Gambling Task

## Abstract

**Background:**

Reward learning is thought to undergo refinement in adolescence, but little is known about how computational components of reinforcement learning develop. Given that adolescence is a sensitive period for reward system plasticity with associated vulnerability for depression, it is important to understand developmental trajectories of different reinforcement learning parameters in normative development and in youth at risk for depression.

**Methods:**

Youth aged 9–17 years completed the Play‐or‐Pass Iowa Gambling Task (PoP‐IGT) across five timepoints. We calculated task metrics using a traditional scoring approach – yielding summary scores for good deck play, bad deck play, and net play – and a computational modeling approach – yielding parameters for reward learning rate, punishment learning rate, go bias, and sensitivity to win/loss frequency ignoring outcome magnitude. We examined normative developmental trajectories for each traditional and computational performance metric using multilevel models. Further, we examined whether maternal history of depression was associated with individual differences in these trajectories.

**Results:**

As hypothesized, youth showed a significant age‐related increase in net play (*p* = 0.003), a measure of overall good performance. Exploratory analyses found that youth showed significant developmental change in reward‐specific learning parameters including age‐related increases in win/loss frequency sensitivity ( *FDR* = 0.016) and age‐related decreases in reward learning rate ( *FDR* < 0.001). In line with hypotheses, youth at high risk for depression showed lower reward learning rates in early adolescence (*p* = 0.041).

**Conclusions:**

The observed developmental changes in traditional and computational metrics are largely consistent with the optimization of learning from rewards across adolescence. Further, the observed developmental changes in specifically reward‐related computational parameters are consistent with heightened adolescent reward system plasticity. Additionally, there was support for our hypothesis that maternal history of depression may exert a unique effect on learning from rewards specifically, but further research across additional reward learning tasks is needed.

## Introduction

The development of reward learning across adolescence is a critical area of research as it can inform models of adolescent motivation, decision‐making, and vulnerability to psychopathology. Multimodal and cross‐species evidence shows the reward system undergoes substantial development across adolescence (Galvan, 2010; Geier & Luna, 2009; Luciana & Collins, 2022; Spear, 2000), with concomitant increases in reward responsivity (Galván, 2013; Shulman et al., 2016; Steinberg et al., 2008; Urošević, Collins, Muetzel, Lim, & Luciana, 2012). However, reward responsivity is multifaceted, and distinct components show different developmental trajectories (Mattoni, Sullivan‐Toole, & Olino, 2024; Urošević et al., 2012). Reward learning is similarly complex, and distinct reward learning components may show heterogeneous development patterns, but little is known about reward learning across development.

Learning from rewards appears to be optimized in adolescence (see Nussenbaum & Hartley, 2019 for review). Developmental research using the Iowa Gambling Task (IGT), a common reward learning task, typically demonstrates linear performance improvements from childhood into young adulthood (Almy, Kuskowski, Malone, Myers, & Luciana, 2018; Cassotti, Houdé, & Moutier, 2011; Crone & van der Molen, 2007; Prencipe et al., 2011; but see also Smith, Xiao, & Bechara, 2012). However, learning from rewards is complex, and distinct subcomponents of reward learning function together to comprise the observable effects that are captured with typical gross summary scores (Haines, Vassileva, & Ahn, 2018; Sullivan‐Toole, Haines, Dale, & Olino, 2022), and different learning subcomponents may show different associations with neurobiological and psychological processes. For example, in a sample aged 10–30 using a version of the IGT with separate measures for approaching reward and avoiding loss, approach increased quadratically following an inverted U‐shaped curve peaking in mid‐to‐late adolescence, while avoidance increased linearly with age (Cauffman et al., 2010). Using the same task in an adolescent sample, puberty predicted approach, while age predicted avoidance (Icenogle et al., 2017). While these studies offer valuable insights into adolescent reward learning, most reward learning studies are limited by restricted measurement strategies.

Reinforcement learning (RL) models expand the range of measures available to examine learning processes beyond typical gross summary scores (e.g., a summary score of points earned). By decomposing reward‐related behavior (e.g., choices) into component parts (e.g., learning rates vs. a general tendency to respond), RL models reveal mechanisms that drive reward response (e.g., Haines et al., 2018). However, little is known about how computational RL components change across development, as most developmental RL studies rely on cross‐sectional samples (Cutler, Apps, & Lockwood, 2022; Nussenbaum & Hartley, 2019). To characterize within‐person RL trajectories, longitudinal designs are necessary (Crone & Elzinga, 2015). Further, modeling RL in longitudinal data allows interrogation of different nuanced components of reward learning that may follow distinct developmental patterns.

Understanding RL development may also offer insights into underlying mechanisms of psychopathology. Adolescence is a sensitive period for reward system development, with heightened reward system plasticity thought to confer increased vulnerability for depression (Davey, Yücel, & Allen, 2008; Lee et al., 2014; Luciana & Collins, 2022; Paus, Keshavan, & Giedd, 2008). For example, blunted reward responsivity in development is a core factor influencing intergenerational transmission of depression (Ethridge et al., 2022; Gotlib, Goodman, & Humphreys, 2020; Kujawa, Hajcak, & Klein, 2019; Pizzagalli, 2022). However, little is known about RL trajectories, either in normative development or in youth at risk for depression.

To understand how RL components change across adolescence and how trajectories might be altered in youth at risk for depression, we used the Play‐or‐Pass Iowa Gambling Task (PoP‐IGT; Case & Olino, 2020; Cauffman et al., 2010; Peters & Slovic, 2000) administered up to five times in youth aged 9–17. The PoP‐IGT allows distinct quantification of approaching reward, avoiding loss, and overall good performance. Additionally, we parsed performance into more fine‐grained learning components using an RL model our group developed for the PoP‐IGT (Haynes, Haines, Sullivan‐Toole, & Olino, 2024), which yields measures of reward learning rate, punishment learning rate, sensitivity to reward/loss frequency ignoring outcome magnitude, and a general tendency to play. We examined developmental trajectories for the traditional and computational learning metrics across normative adolescence. Additionally, we also examined whether a maternal history of depression, a robust risk factor for depression (Jacobs, Talati, Wickramaratne, & Warner, 2015; Klein, Lewinsohn, Rohde, Seeley, & Olino, 2005), was associated with altered trajectories. We controlled for factors known to affect IGT performance including cognitive functioning (Almy et al., 2018; Cauffman et al., 2010; Gansler, Jerram, Vannorsdall, & Schretlen, 2011; Icenogle et al., 2017), and sex (van den Bos, Homberg, & de Visser, 2013; Zanini, Picano, & Spitoni, 2024).

In line with general predictions of optimized reward learning across adolescence (Nussenbaum & Hartley, 2019), we hypothesized that good deck proportion play and net proportion play (a measure of overall good performance) would increase longitudinally across adolescence while bad deck proportion play would decrease. We also expected to see age‐related change in reinforcement learning parameters but made no directional hypotheses. Additionally, given that blunted reward responsivity is thought to be a mechanism of intergenerational transmission of depression, we hypothesized that good proportion play and reward learning rate, specifically, would be relatively reduced in youth with a maternal history of depression, but made no specific predictions related to associations between other performance metrics and maternal history of depression.

## Method

### Participants

Participants were drawn from the Temple Adolescent Development Study, a prospective longitudinal study of reward function development. This study was approved by the Institutional Review Board at Temple University and performed in accordance with the Declaration of Helsinki. English‐speaking youth aged 9–14 years who had at least one biological parent living in the home were invited to participate and assessed across 36 months. Exclusion criteria included child or parental history of bipolar disorder or psychotic spectrum disorder and child diagnosis of serious neurological illness, head injury, learning disabilities, or developmental disabilities, including autism spectrum disorders. General intellectual function was assessed at baseline using the Kaufman Brief Intelligence Test (K‐BIT‐2; Kaufman & Kaufman, 2004), and participants with an IQ falling two standard deviations or more below the mean (full scale IQ < 70) were excluded from participation. Parents provided written informed consent for their child to participate, and youths provided written assent. The current data included data from youth 9–17 years old completing the PoP‐IGT five times, spaced approximately nine months apart (T1 *n* = 208, T2 *n* = 157, T3 *n* = 135, T4 *n* = 98, T5 *n* = 73). At baseline, the sample was comprised of 9–14‐year‐olds (mean age at baseline = 10.98 (*1.48*)), 59% female youth, and of the 79% of the sample that reported race, 41% were White, 44% were Black, 1% were Asian, 12% were multiracial, and overall 59% reported a racial minority status. See Table 1 for baseline demographic information and Table S1 for demographic information across additional timepoints.

**Table 1:** Youth with and without a maternal history of depression were comparable on baseline demographics

	Timepoint 1 (*n* = 208)				
	Maternal Hx of depression (*n* = 74)	No maternal Hx of depression (*n* = 134)	*X* ^2^	*t*‐stat	*p*‐Value
	Mean (*SD*) or frequency				
Age	10.75 (*1.49*)	11.11 (*1.47*)		−1.66	.098
Sex (female)	61%	57%	0.10		.747
K‐BIT nonverbal IQ	103.86 (*14.50*)	100.69 (*15.20*)		1.48	.140
In‐lab administration/E‐prime	100%	100%	NA		NA
Racial minority	48% (of 70% reporting race)	63% (of 84% reporting race)	2.83		.093
	**Racial breakdown among those reporting race**				
White	52%	37%			
Black	40%	46%			
Asian		1%			
Multiracial	6%	15%			
Other	2%	2%			

See Table S1 for demographic information at all five timepoints.

### Play‐or‐Pass Iowa Gambling Task (PoP‐IGT)

All task outcomes were hypothetical. Participants began the task with a ‘bank’ of $2,000 and were shown that the task involved four decks of cards. On each trial, a single deck was presented, and participants had the opportunity to ‘play’ or ‘pass’ on the presented deck. If a participant played on a deck, they would receive either a monetary gain, a loss, or neither (i.e., $0 change) from the drawn card. If a participant passed on a deck, they moved onto the next trial. Each deck was associated with a different payout distribution such that two decks yielded a net loss across the task (i.e., the disadvantageous/bad decks) and two decks yielded a net gain across the task (i.e., the advantageous/good decks). There were two versions of the task with different mappings of decks to payout structures, and task versions were randomized across task administrations to preserve trial‐and‐error learning and reduce practice effects due to recognition of decks. For analyses, all decks were recoded such that Decks A and B were the net loss decks and Decks C and D were the net gain decks. The order of deck presentation (i.e., decks participants were offered to play or pass on) and the sequence of outcomes associated with each deck was fixed across participants. Participants were not informed of the payout distribution associated with each deck nor the sequence of decks that would be presented and had to learn about the outcomes associated with each deck through sampling the decks. Trials ended if participants did not respond within four seconds and these trials were coded as a ‘pass’ trial. Finally, participants were told that their task earnings would be exchanged for a real cash bonus; however, all participants received $10 upon task completion regardless of performance.

### PoP‐IGT task administration

The PoP‐IGT was primarily administered in‐lab using E‐Prime Stimulus Presentation Software (Schneider, Eschman, Zuccolotto, & Burgess, 2002). Due to the COVID‐19 pandemic, approximately 11% and 59% of participants' data at timepoint 4 and timepoint 5, respectively, were collected remotely using PsychoPy and Pavlovia (Bridges, Pitiot, MacAskill, & Peirce, 2020; Peirce et al., 2019; see Table S1 for further details). A binary variable for in‐lab task administration via E‐Prime was therefore included as a covariate in the longitudinal models.

### Structured clinical interview for DSM disorders (SCID)

Mothers were interviewed with the Structured Clinical Interview for DSM‐V (SCID‐V‐RV; First, Williams, Karg, & Spitzer, 2015) to assess clinical diagnoses of various mental disorders. Maternal history of depression was operationalized as a lifetime history of major depression or persistent depression. At the baseline assessment, maternal history of depression was present in 36% (*n* = 74) of mothers.

### Kaufman Brief Intelligence Test (K‐BIT) nonverbal IQ

Youth completed the Kaufman Brief Intelligence Test (K‐BIT‐2; Kaufman & Kaufman, 2004) to assess cognitive function. The standardized nonverbal cognitive function score was used as a covariate in the current analyses.

### Child Behavior Checklist (CBCL)

At baseline, mothers completed the Child Behavior Checklist (CBCL; Achenbach, 2009) to provide dimensional assessments of their child's current behavioral and emotional problems. To quantify the child's own internalizing symptoms for an additional exploratory analysis, we used the internalizing scale, including 32 items reflecting anxiety and depression symptoms. CBCL data were available for 152 youth.

### Data analysis

#### Data analysis overview

Across each of the five assessments, we calculated behavioral metrics from the PoP‐IGT data using a traditional scoring approach and a computational modeling approach. General statistical analyses and longitudinal analyses were conducted in R (v. 4.2.2; R Core Team, 2022). The computational model used hierarchical Bayesian analysis (HBA) computed in Stan (v. 2.32; Stan Development Team, 2023a, 2023b), a probabilistic programming language, which estimates parameters using Hamiltonian Monte Carlo, a variant of Markov chain Monte Carlo (MCMC) to sample from high‐dimensional probabilistic models. We used the RStan package (v. 2.26.22; Stan Development Team, 2023a, 2023b) to interface between R and Stan.

#### Traditional summary statistic scoring of PoP‐IGT

Traditional scoring involved calculating the proportion of plays on good (i.e., advantageous) decks and bad (i.e., disadvantageous) decks, separately at each timepoint. Session‐wide play proportions on good and bad decks represent gross measures of reward and punishment learning, respectively. Specifically, more plays on the good decks reflect higher reward learning (i.e., approach of reward) and fewer plays on the bad decks reflect higher punishment learning (i.e., avoidance of punishment). Additionally, a net score was computed (good deck choices minus bad deck choices), with the net score representing a gross measure of overall good decision‐making on the task. These summary scores were used as outcome variables in the multilevel models. Test–retest reliabilities for traditional summary statistics from assessments spaced nine months apart were in the poor‐to‐moderate range for the first three assessments and showed good reliability between the final two assessments (see Table S2).

#### Computational modeling of PoP‐IGT

Computational parameters were estimated with hierarchical Bayesian analysis using a modified version of the Outcome‐Representation Learning (ORL) model (Haines et al., 2018). Specifically, the PoP‐ORL model was developed by Haynes and colleagues to dissociate decision‐making mechanisms driving choice behavior in the context of the PoP‐IGT (Haynes et al., 2024). Haynes and colleagues showed that decision‐making on the PoP‐IGT was best explained using four free parameters: where reward learning rate (*A*_rew_) describes updating value estimates in response to gains, punishment learning rate (*A*_pun_) describes updating value estimates in response to losses, go bias (β*b*) describes a general tendency to play (rather than pass) across all decks, and win/loss frequency sensitivity (β*f*) describes updating value estimates in response to the frequency of wins and losses. While we refer to β*f* simply as ‘win/loss frequency sensitivity’ throughout for brevity, it is critical to note that this parameter reflects sensitivity to the frequency of rewarding and punishing outcomes while ignoring outcome magnitude. The PoP‐ORL model was fit separately at each timepoint.

The model yielded posterior distributions for each of the four free parameters. Posterior means for each of the four parameters were extracted for each individual participant for each of the five timepoints of task data and used as outcome variables in the multilevel models. As the PoP‐ORL model yielded learning rates that were bounded between 0 and 1, the *A*_rew_ and *A*_pun_ parameter means were normalized per the inverse cumulative distribution function of the standard normal distribution (i.e. the ‘qnorm’ function in R) before being used as outcome variables. Test–retest reliabilities for the computational parameters from assessments spaced nine months apart were in the poor range for the first three assessments and showed moderate reliability between the final two assessments (see Table S2).

#### Summary of Play‐or‐Pass ORL (PoP‐ORL) model

See Haynes et al. (2024) for full model details. To summarize the PoP‐ORL model, choices to play or pass were modeled as a function of the value of playing on a given deck using the following logistic function:

$$Y_{j}\left(t\right)\sim \text{bernoulli}\;\left(\frac{1}{1+\exp\left(-V_{j}\left(t\right)\right)}\right)$$

where $Y_{j}\left(t\right)$ indicates whether the participant played ($Y_{j}\left(t\right)$ = 1) versus passed ($Y_{j}\left(t\right)$ = 0) when presented with deck *j* on trial *t*. While the value of passing is held constant at 0, the value of playing is updated such that the expected value (*EV*) of outcomes, expected frequency (*EF*) of outcomes, and a bias for playing (β*b*) are integrated linearly to generate a value signal for each deck *j* on trial *t*, as follows:

$$V_{j}\left(t+1\right)={\mathrm{EV}}_{j}\left(t+1\right)+{\mathrm{EF}}_{j}\left(t+1\right)\cdot \beta f+\beta b$$

**Figure 1:**
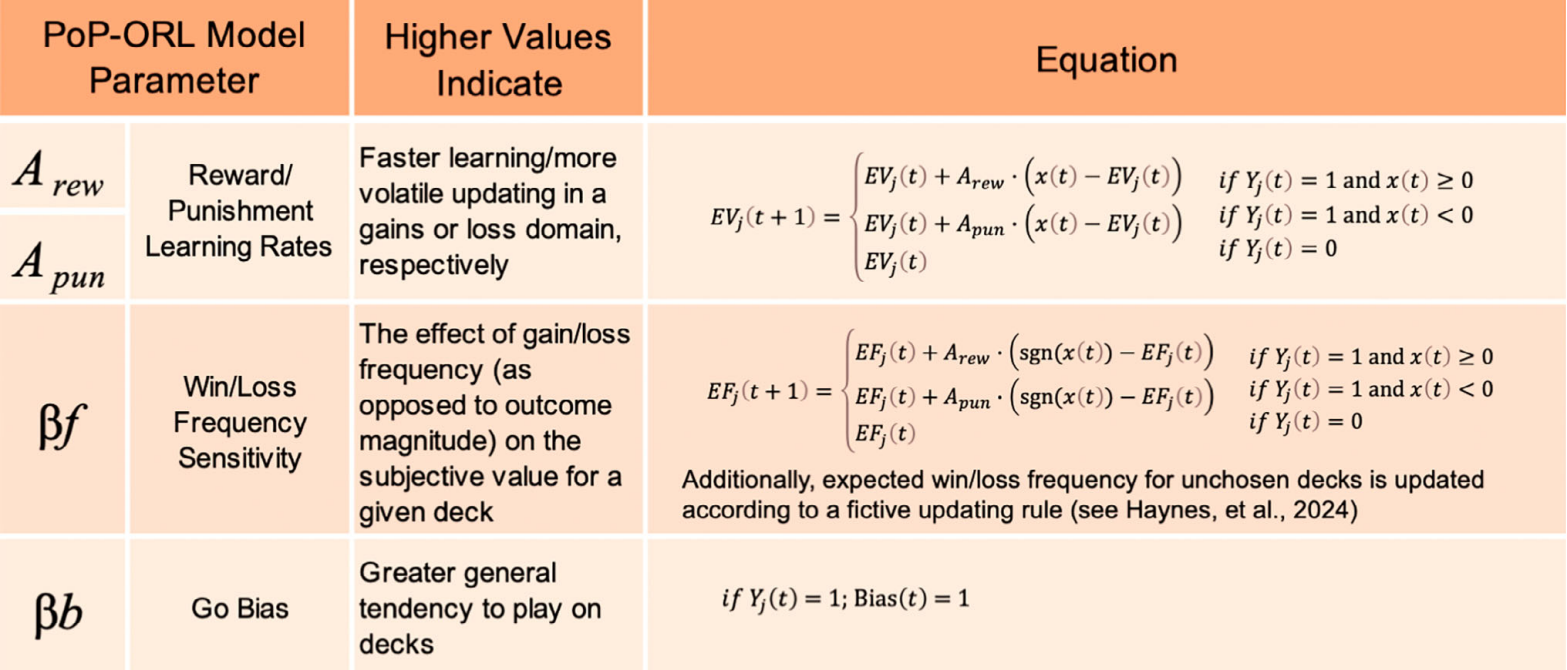
Summary of the PoP‐ORL model parameters. Four free parameters – reward learning rate, punishment learning rate, win/loss frequency sensitivity ignoring outcome magnitude, and go bias – inform a trial‐by‐trial value signal for each deck. See Haynes et al. (2024) for full details of the adapted PoP‐ORL model

The four free parameters that inform this value signal are summarized in Figure 1.

#### PoP‐ORL model validation

Model validation included both parameter recovery and posterior predictive checks. Parameter recovery was generally in the good‐to‐excellent range across the timepoints for all parameters except for *A*_rew_ where parameter recovery correlations were in the range of 0.59–0.67 (see Figure S1; see also Haynes et al., 2024). The reward‐specific recovered parameters, *A*_rew_ and β*f*, showed weak or no correlation with one another (see Figure S2), suggesting independence of these parameters and their respective developmental trajectories. Posterior predictive checks demonstrated that the model‐simulated data was a good fit to the observed data (see Figure S3).

#### Assessing test–retest reliability of performance metrics

Generally similar to the reliabilities previously reported in adults tested at a one‐month interval (Haynes et al., 2024), our youth sample tested at approximately nine‐month intervals showed test–retest reliabilities in the poor‐to‐fair range across the first three timepoints for both the PoP‐IGT traditional metrics (*r* = .36–.54) and computational parameters derived from the PoP‐IGT model (*r* = .11–.48). Across the final two timepoints, test–retest reliabilities had improved to the fair‐to‐good range for the PoP‐IGT traditional metrics (*r* = .69–.75) and the computational parameters (*r* = .51–.58) except for *A*_rew_, which showed poor reliability between the final two timepoints (*r* = .36; see Table S2).

#### Assessing associations between PoP‐ORL parameters and overall task performance

As the computational parameters describe behavior thought to drive the gross measures of reward learning captured by traditional summary scores, we examined associations between the PoP‐ORL parameters and ‘net proportion play’ (representative of overall good performance) to understand how parameters were impacting performance at different timepoints and to aid in the interpretation of changes in parameters across time. As these analyses were exploratory, false discovery rate (*FDR*; Benjamini & Hochberg, 1995) correction was applied to the set of correlations at each timepoint.

#### Examining performance metric developmental trajectories and depression risk as a predictor of performance trajectories

To understand change over time in all performance measures, longitudinal behavioral metrics from the traditional and computational analyses were entered into a series of multilevel models, using the lme4 package (Bates, Mächler, Bolker, & Walker, 2015). Nested models were compared using likelihood ratio tests. Across the models, ‘baseline effects’ refer to between‐group differences at 10.98 years old, the average age at timepoint 1 (age was centered at the average baseline age), and ‘age‐related effects’, or ‘trajectories’, refer to the rate of change across age (slope over time). Each set of longitudinal behavioral metrics was separately entered into the following series of multilevel models. First, unconditional linear growth models estimated trajectories (model 1) to examine linear change in the metric across age. Second, unconditional quadratic growth models estimated trajectories (model 2) to examine nonlinear change in the metric across age. Next, linear and quadratic models were compared for the respective behavioral metric, and the model with the best fit (based on a significant likelihood ratio test) was then used for conditional growth models. Conditional growth models (model 3) examined maternal history of depression as a predictor of the performance metric growth trajectories (interaction of age and risk group), building on the winning unconditional model (linear or quadratic). Finally, covariates including child sex, K‐BIT nonverbal IQ, and task administration modality (in‐lab/E‐prime vs. remote/Pavlovia) were added to the conditional growth models (model 4). Coefficients from model 4 are reported in text for each performance metric in order to make models comparable across performance metrics and because model 4 included all planned covariates. An additional exploratory analysis tested whether the child's own baseline internalizing symptoms accounted for variance in performance metrics significantly impacted by maternal history of depression, above‐and‐beyond maternal history, by additionally testing the interaction of age and CBCL child internalizing.

#### Significance thresholds

For a priori hypotheses, a significance threshold of *p* < .05 was used. Analyses testing a priori directional hypotheses included: (i) testing age‐related change in the traditional metrics, and (ii) testing reductions in good proportion play and reward learning rate in high‐risk youth. For exploratory analyses, false discovery rate (*FDR*; Benjamini & Hochberg, 1995) was applied to correct for multiple comparisons, and the threshold for significance was set at *FDR* < 0.05. Exploratory analyses subjected to *FDR* correction included: (i) testing associations between the PoP‐ORL parameters and net proportion play, and (ii) testing age‐related change in the computational parameters (as no directional effects were hypothesized for computational parameters). Of note, the PoP‐ORL model was estimated using HBA, and the model did not condition on the age or depression‐risk covariates. As such, the HBA model is likely to compress true between‐subject variation that is related to these covariates, due to ‘shrinkage’ (Katahira, 2016). In the subsequent multilevel model, regression coefficients may therefore be attenuated, producing smaller effect sizes for these covariates. Thus, estimates for the effects of age and depression‐risk are likely conservative.

## Results

### Increased punishment learning rate and win/loss frequency sensitivity support overall good performance

Youth with and without a maternal history of depression showed similar associations between PoP‐ORL parameters and overall good performance as measured by net proportion play across the five assessments, suggesting the learning parameters function similarly to support task performance across both groups (see Figure S4). Across the entire sample, punishment learning rate and sensitivity to win/loss frequency showed significant and generally strong positive correlations with net proportion play across all timepoints (*r* = .37–.72, *FDR* < 0.001). Reward learning rate showed a nonsignificant negative correlation with net proportion play only at timepoint 1 (*r* = −.15, *FDR* = 0.054), nonsignificant associations at timepoint 2 (*r* = −.09, *FDR* > 0.05) and timepoint 4 (*r* = .16, *FDR* > 0.05), and significant positive associations at timepoint 3 (*r* = .26, *FDR* = 0.014) and timepoint 5 (*r* = .31, *FDR* = 0.017), suggesting that reward learning rate may play a different role in PoP‐IGT performance across multiple assessments. Go bias showed weak and nonsignificant associations with net proportion play at timepoint 1 (*r* = .09, *FDR* > 0.05) and timepoint 2 (*r* = .09, *FDR* > 0.05), and significant positive associations at timepoints 3–5 (*r* = .22–.42, *FDR* ≤ 0.01).

### Overall good performance increased across adolescence

**Figure 2:**
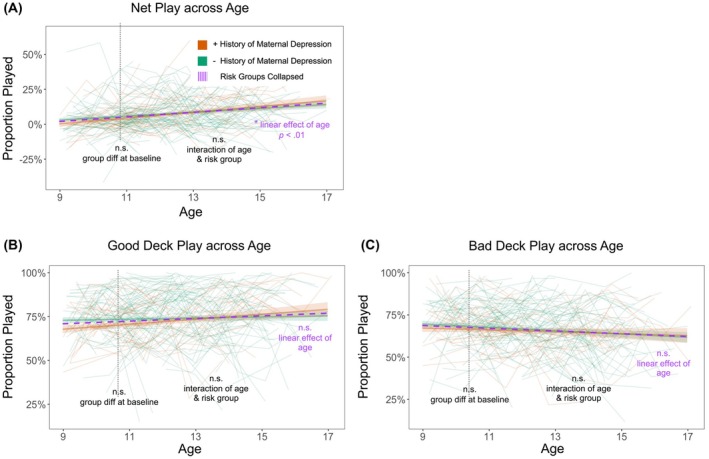
Across development, adolescents show improved overall performance on the PoP‐IGT. Developmental trajectories for each traditional summary score, based on conditional growth models including all covariates (model 4), showed, as hypothesized, (a) a significant age‐related increase in net proportion play, a measure of overall good performance (*p* < .01). However, there were no significant effects of age for (b) approaching good decks or (c) avoiding bad decks. In line with hypotheses, youth with a maternal history of depression showed a lower proportion of good plays at baseline, but this difference was not significant (*p* = .051)

As hypothesized, net proportion play, a measure of overall good performance, showed significant age‐related increases (*b* = 0.01, *SE* = 0.00, *t*(661) = 2.97, *p* = .003; Figure 2a). However, contrary to hypotheses, age‐related changes in approaching rewards (good proportion play; Figure 2b) and avoiding punishment (bad proportion play; Figure 2c) were not significant (*p* > .05). For all traditional summary score metrics, quadratic age effects did not explain more variance than linear effects of age. Higher cognitive ability was associated with better performance at baseline (average age of ~11 years old) across all three traditional performance metrics (*p* < .01). Child sex and on‐site administration were not significant predictors of traditional performance metrics.

### Reward‐related learning parameters, but not punishment learning rate, showed significant change across adolescence

**Figure 3:**
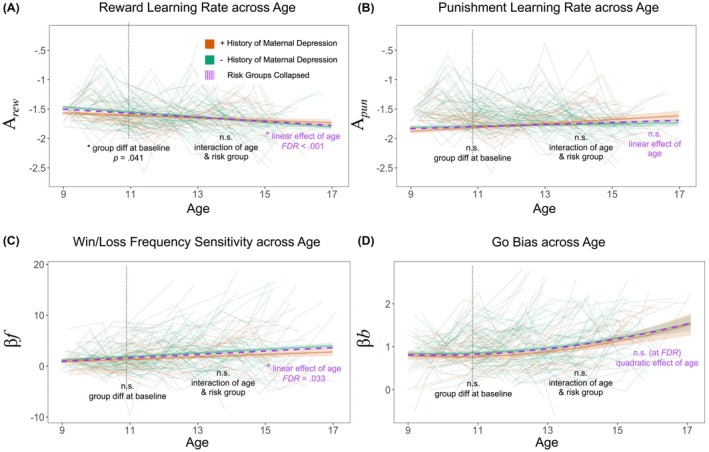
Reward‐related learning parameters show significant change across adolescence. Developmental trajectories for each ORL parameter mean, based on conditional growth models including all covariates (model 4), showed (a) a significant age‐related linear decrease in reward learning rate (*FDR* < 0.001) and (c) a significant linear increase in sensitivity to win/loss frequency ignoring outcome magnitude (*FDR* < 0.005). However, (b) punishment learning rate did not show significant age‐related change, and (d) for go bias, both the linear and quadratic effects of age were only significant in models 2 and 3 but were not significant in the full model 4 (*FDR* < 0.005). In line with hypotheses, maternal history of depression only impacted reward learning rate, with at‐risk youth showing reduced reward learning rates at the average baseline age of ~11 years old (*p* = .041)

Interestingly, only the two reward‐related learning parameters, reward learning rate and sensitivity to win/loss frequency ignoring outcome magnitude, showed significant developmental change across adolescence. Reward learning rate showed a significant linear decrease (*b* = −0.04, *SE* = 0.01, *t*(661) = −5.49, *FDR* < 0.001; Figure 3a). Win/loss frequency sensitivity showed a significant linear increase (*b* = 0.31, *SE* = 0.13, *t*(661) = 2.41, *FDR* = 0.016; Figure 3c). In contrast to reward learning rate, punishment learning rate (Figure 3b) showed a subtle and nonsignificant developmental increase (*FDR* > 0.05). Go bias (Figure 3d) showed significant positive linear and quadratic age‐related increases only in models 2 and 3 (model 3 *p* < .05); however, when all covariates were added to the full model (model 4), only the quadratic effect of age was significant (*p* < .05), but only at an uncorrected threshold (*FDR* > 0.05). Higher cognitive ability was associated with higher punishment learning rate and win/loss frequency sensitivity at the average baseline age (*p* < .05) but did not show significant associations with reward learning rate or go bias (*p* > .05). Child sex was not a significant predictor of the PoP‐ORL parameters. On occasions with on‐site administration, youth had higher reward learning rates, lower win/loss frequency sensitivity, and lower go bias (*p* < .01), with no effect on punishment learning rate. For all metrics (traditional scores and PoP‐ORL parameters), results of the full conditional growth models with all covariates (model 4) are shown in Table 2 (see Table S3 for results across all nested models).

**Table 2:** Results from full conditional growth models including all covariates (model 4) for each traditional summary score and ORL parameter mean

	Traditional scoring (proportion played)			Computational modeling (model‐derived parameter means)			
	Net (good – bad)	Good deck	Bad deck	*A*_rew_	*A*_pun_	β*f*	β*b*
Fixed effects							
Constant	0.08*** [0.03 to 0.12]	0.80*** [0.75 to 0.85]	0.72*** [0.67 to 0.77]	−1.67*** [−1.76 to −1.59]	−1.81*** [−1.91 to −1.71]	4.42*** [3.25 to 5.59]	1.25*** [1.08 to 1.43]
Age	0.01** [0.00 to 0.02]	0.00 [−0.01 to 0.01]	−0.01 [−0.02 to 0.00]	−0.04*** [−0.06 to −0.03]	0.01 [−0.01 to 0.03]	0.31* [0.06 to 0.56]	0.03 [−0.01 to 0.08]
Age^2^	NA	NA	NA	NA	NA	NA	0.01* [−0.00 to 0.03]
Maternal Hx depression	−0.01 [−0.04 to 0.01]	−0.03 [−0.06 to 0.00]	−0.02 [−0.05 to 0.02]	−0.06* [−0.11 to −0.00]	−0.02 [−0.08 to 0.04]	−0.52 [−1.21 to 0.17]	−0.08 [−0.20 to 0.03]
Age × maternal Hx depression	0.01 [−0.01 to 0.02]	0.01 [−0.01 to 0.03]	0.00 [−0.01 to 0.02]	0.02 [−0.00 to 0.05]	0.02 [−0.01 to 0.06]	0.04 [−0.39 to 0.47]	−0.01 [−0.09 to 0.07]
Age^2^ × maternal Hx depression	NA	NA	NA	NA	NA	NA	0.00 [−0.02 to 0.03]
K‐BIT nonverbal standard IQ	0.00*** [0.00 to 0.00]	0.00** [0.00 to 0.00]	−0.00** [−0.00 to −0.00]	0.00 [−0.00 to 0.00]	0.01*** [0.00 to 0.01]	0.05*** [0.03 to 0.07]	0.00 [0.00 to 0.01]
Child sex	0.00 [−0.03 to 0.03]	−0.03 [−0.06 to 0.01]	−0.03 [−0.06 to 0.00]	0.00 [−0.04 to 0.05]	0.01 [−0.05 to 0.07]	−0.10 [−0.75 to 0.56]	−0.06 [−0.16 to 0.04]
On‐site administration	−0.02 [−0.06 to 0.02]	−0.04 [−0.08 to 0.00]	−0.01 [−0.05 to 0.03]	0.12** [0.04 to 0.20]	0.01 [−0.08 to 0.09]	−2.50*** [−3.51 to −1.48]	−0.33*** [−0.48 to −0.19]
Random effects							
σ^2^	0.01	0.01	0.01	0.06	0.06	8.15	0.17
τ_00 id_	0.00	0.01	0.01	0.01	0.01	1.21	0.05
τ_11 id.age_	0.00	0.00	0.00	0.00	0.00	0.75	0.01
Model comparison (unconditional growth model is reference)	$X_{5}^{2}$ = 38.99***	$X_{5}^{2}$ = 13.42*	$X_{5}^{2}$ = 12.63*	$X_{5}^{2}$ = 15.65**	$X_{5}^{2}$ = 31.23***	$X_{5}^{2}$ = 42.99***	$X_{6}^{2}$ = 22.88***
ICC	0.21	0.32	0.36	0.12	0.15	0.13	0.22
Pseudo *R* ^2^ (total)	.34	.36	.39	.20	.24	.26	.35

Reported coefficients are unstandardized and 95% confidence intervals are represented in brackets. See Table S3 for results across full nested models.

* *p* < .05.

** *p* < .01.

*** *p* < .001.

### Maternal history of depression is associated with lower reward learning rate in early adolescents

For the traditional summary scores, there were no significant performance differences between depression‐risk groups at either baseline or in the age‐related trajectories (*p* > .05). In line with our hypothesis, youth with a maternal history of depression showed a lower proportion of good plays at baseline, but this difference was not significant (*b* = −0.03, *SE* = 0.02, *t*(661) = −1.69, *p* = .092; Figure 2b). Maternal history of depression had a specific impact on reward learning rate, with the at‐risk youth showing reduced reward learning rates at the average baseline age of ~11 years old (*b* = −0.06, *SE* = 0.03, *t*(661) = −2.04, *p* = .041; Figure 3a). The interaction of maternal history of depression and reward learning rate trajectory was not significant (*p* > .05). The child's own baseline internalizing symptoms did not account for additional variance in reward learning rates beyond the maternal history predictor (*p* > .05).

## Discussion

Our results support a general optimization of reward learning during adolescence (Afshar, Keip, Taylor, Lee, & Groman, 2020; Davidow, Foerde, Galván, & Shohamy, 2016; Eckstein, Master, Dahl, Wilbrecht, & Collins, 2022; Nussenbaum & Hartley, 2019; Palminteri, Kilford, Coricelli, & Blakemore, 2016; Xia et al., 2021), corresponding to age‐related improvements in net play across successive administrations of the reward learning task. Contrary to our hypotheses, traditional metrics provided no evidence for age‐related change in approaching rewards or avoiding punishments. This finding is inconsistent with previous cross‐sectional research using the PoP‐IGT (Cauffman et al., 2010; Icenogle et al., 2017) and suggests these processes may be nuanced and not explained by age alone (Icenogle et al., 2017). A major goal of our study was to map developmental trajectories for different RL parameters across adolescence, and consistent with expectations, we found evidence for adolescent development of different RL components. Interestingly, there was only significant age‐related change in reward‐specific learning parameters. Across adolescence, reward learning rate decreased while sensitivity to win/loss frequency ignoring outcome magnitude increased. In contrast, change in punishment learning rate and the general tendency to play were not significant in the full models. Adolescent change in reward‐specific learning parameters is consistent with robust evidence demonstrating that adolescence is a time of heightened reward system plasticity (Galvan, 2010; Geier & Luna, 2009; Luciana & Collins, 2022; Spear, 2000), and consistent with emerging evidence that reward learning may peak in adolescence (Eckstein et al., 2022; Palminteri et al., 2016).

Adolescent decreases in reward learning rate may seem to contradict the well‐established developmental model of heightened adolescent reward responsiveness (Casey, Getz, & Galván, 2008; Galván, 2013; Luciana & Collins, 2022; Shulman et al., 2016; Steinberg et al., 2008); however, this finding should be interpreted in light of evidence that learning parameters are highly influenced by task demands, and that optimal learning rates depend on task structure (Bishop & Gagne, 2018; Davidow et al., 2016; Eckstein, Wilbrecht, & Collins, 2021; Nussenbaum & Hartley, 2019). Across different tasks, evidence for directional developmental change in learning rates is mixed (Cutler et al., 2022; Nussenbaum & Hartley, 2019), with evidence of developmental increases in learning rate (Master et al., 2020; Xia et al., 2021) and decreases in learning rate (Decker, Lourenco, Doll, & Hartley, 2015). Our results also provide mixed evidence for the direction of change in learning rates across adolescence. While reward learning rate showed age‐related decreases, punishment learning rate showed subtle and nonsignificant age‐related increases, suggesting that learning rates are fluid and context‐dependent rather than fixed (Bishop & Gagne, 2018; Davidow et al., 2016; Eckstein et al., 2021). In the IGT, rewards can distract from infrequent but high‐magnitude losses (Chiu et al., 2008; Haines et al., 2018). This task feature, coupled with putative refinement of reward learning in adolescence (Nussenbaum & Hartley, 2019), suggests age‐related reductions in reward learning rate on the PoP‐IGT likely reflect developmental optimization of this learning parameter in service of task performance.

Similarly, developmental increases in win/loss frequency sensitivity likely reflect developmental tuning of this specific parameter, enabling task proficiency. This interpretation aligns with our finding that higher win/loss frequency sensitivity was consistently associated with better task performance at each timepoint. Notably, the PoP‐ORL model win/loss frequency parameter reflects sensitivity to the frequency of rewards and losses *ignoring outcome magnitude* (Haines et al., 2018; Sullivan‐Toole et al., 2022). Thus, increases in win/loss frequency sensitivity across adolescence could reflect a general increase in sensitivity to reward/loss frequency or could also reflect increases in the ability to selectively ignore reward magnitude and attend to other features of the environment in service of a goal (Decker, Otto, Daw, & Hartley, 2016; e.g., not being distracted by Deck A's high‐magnitude rewards is beneficial as this deck yields a net loss). Overall, our results support a model whereby mechanisms of reinforcement learning, and particularly mechanisms related to learning from rewards specifically, are refined across adolescence to facilitate effective engagement with the environment.

This study also provides tentative support for reduced reward learning in youth with a maternal history of depression, aligning with substantial evidence of blunted reward responsivity in youth with familial depression risk (Abitante, Haraden, Pine, Cole, & Garber, 2022; Bruder‐Costello et al., 2007; Durbin, Klein, Hayden, Buckley, & Moerk, 2005; Gotlib et al., 2010; Kujawa & Burkhouse, 2017; McCabe, Woffindale, Harmer, & Cowen, 2012; Monk et al., 2008; Olino et al., 2011, 2014; Rawal, Collishaw, Thapar, & Rice, 2013) as well as emerging evidence that reward learning, specifically, may be blunted in youth with familial risk (Belleau et al., 2021; but see also Morris, Bylsma, Yaroslavsky, Kovacs, & Rottenberg, 2015). We hypothesized that both good proportion play and reward learning rate would be lower in high‐risk youth, and there was some evidence for reductions in both during early adolescence. While the high‐risk early adolescents in our sample showed significantly reduced reward learning rates, the high‐risk group showed only a nonsignificant trend toward reduced plays from good decks in early adolescence. As the interactions between risk group and these two reward metric trajectories were not significant, results suggest that reward learning may be generally lower for youth with a maternal history of depression across adolescence rather than this effect being restricted to early adolescence. A pattern of blunted reward learning in youth at risk for depression is consistent with research demonstrating attenuations in different forms of reward responsivity in youth at risk for depression (Abitante et al., 2022; Bruder‐Costello et al., 2007; Durbin et al., 2005; Gotlib et al., 2010; Kujawa & Burkhouse, 2017; McCabe et al., 2012; Monk et al., 2008; Olino et al., 2011, 2014; Rawal et al., 2013); however, replication of blunted *reward learning* in at‐risk youth is needed. Even so, our observation of depression risk only being related to differences in a computational reward learning parameter, but not any summary score metrics, aligns with our recent work showing that application of the ORL model to the IGT enhances the task's validity for characterizing reward learning alterations in depression compared to traditional task metrics (Sullivan‐Toole et al., 2022).

This study has several notable strengths including use of the PoP‐IGT, a version of the IGT that allows independent assessment of approaching reward and avoiding punishment, in line with neurobiological and behavioral evidence that these are dissociable learning systems (Christakou et al., 2013; Frank, Seeberger, & O'Reilly, 2004; Gershman, 2015). In addition to enhanced task design, we employed a computational model designed specifically for the PoP‐IGT (Haynes et al., 2024), which yields estimates of distinct latent processes underlying observable learning effects. Finally, we employed a longitudinal design across five waves of data in a moderately large sample of youth, allowing within‐person longitudinal modeling of reward learning trajectories, which has rarely been used in developmental research with the IGT (Almy et al., 2018) or in the context of reinforcement learning (Cutler et al., 2022; Nussenbaum & Hartley, 2019). Further, this study is distinctive in that it tracks reinforcement learning trajectories longitudinally across a substantial portion of adolescence in both normative and at‐risk development – critical for understanding how alterations to reward learning during the adolescent sensitive period may contribute to depression onset.

Current results should also be considered alongside several limitations. First, when using repeated administrations of a task to assess reward learning, it is inherently difficult to disentangle true developmental changes in reinforcement learning from practice effects produced by multiple task engagements (e.g., recollection of task structure). While it may not be possible to fully dissociate developmental changes in learning from practice effects, the current study incorporated several methodological approaches to address this challenge including the use of multiple versions of the task, an accelerated longitudinal design, and multilevel growth modeling. Random assignment of task versions across administrations sought to preserve trial‐and‐error learning and reduce performance effects due to the recognition of decks. The accelerated longitudinal design was implemented by assessing two overlapping developmental cohorts (recruited at either 9–10 years old or 12–13 years old) five times across three years, experimentally decoupling task exposures from age. This design feature coupled with multilevel growth modeling that estimated within‐person age‐related change over time allows examination of developmental trajectories while controlling for practice or memory effects. Future work could further compare longitudinal RL trajectories in additional age ranges to further isolate true developmental change from practice effects.

Second, while our use of a multilevel growth model produced age‐related trajectories for each computational parameter and mitigated measurement error via hierarchical pooling (i.e., shrinkage) across repeated measures assumed to be related (Brown, Chen, Gillan, & Price, 2020; Haines et al., 2025; Haines, Sullivan‐Toole, & Olino, 2023; Matzke et al., 2017), we fit the multilevel growth model to point estimates (i.e., person‐level posterior means) of the computational parameters at each timepoint. This approach is limited because the point estimates are treated as true scores in the multilevel model, rather than estimates with measurement error as they are in joint hierarchical Bayesian models (Haines et al., 2025; Haynes et al., 2024; Sullivan‐Toole et al., 2022). Future work could improve upon this modeling strategy by simultaneously estimating trial‐level learning parameters alongside growth trajectories to allow uncertainty in the computational parameters to inform estimation of the growth parameters – an effort that would represent a significant methodological advance.

Third, the COVID‐19 pandemic occurred toward the end of the study, requiring transition from in‐lab to remote online data collection. Task administration modality was included as a covariate, and while it did not show an effect on the traditional metrics, several learning parameters were associated with on‐site administration. This may obscure developmental effects to some extent, and thus replication is necessary. Additionally, there was attrition across the study, likely biasing results toward families with resources to continue in the study. While attrition and associated biases are an issue in all longitudinal studies, the COVID‐19 pandemic likely exacerbated this issue, further necessitating replication.

Another limitation is that the current study utilized a flat $10 bonus payment irrespective of actual participant performance. This lack of monetary incentivization of good performance may have suppressed individual differences in learning parameters. Finally, while the current data did not allow investigation of the effects of additional relevant predictors such as puberty and youth psychotropic medication use, future work should examine the effects of such covariates on the development of reinforcement learning parameters.

Overall, this study supports a model of reward learning optimization across adolescence, with particular support for adolescent developmental tuning of reward‐specific computational parameters, in line with putative heightened reward system plasticity during adolescence. Further, we found evidence that maternal history of depression may specifically blunt reward learning during early adolescence. These findings help lay the groundwork for investigating how mechanisms of reward learning may be disrupted in youth who go on to develop depression.

## Ethical considerations

As all youth participants were younger than 18 years, data collection procedures only commenced after parents provided consent for participation and youth provided written assent for participation. The research protocols and consent and assent forms were approved by the IRB of Temple University under protocol number 23174, which was reapproved on 8/04/2021.

**Key points**

**What is known?**

- Adolescence is a sensitive period for reward system plasticity, with an associated developmental vulnerability for depression.
- Relatively little is known about how reinforcement learning components develop.
- Reinforcement learning components may show different developmental trajectories across normative adolescence and in youth at risk for depression.

**What is new?**

- Adolescents showed significant age‐related changes in reward, but not punishment, learning parameters.
- Results are consistent with an optimization of learning from rewards across adolescence.
- As hypothesized, youth at high risk for depression showed lower reward learning rates, with the difference evident specifically in early adolescence.

**What is relevant?**

- This work can inform future research and the development of clinical interventions to target specific reward learning processes that may be related to vulnerability to psychopathology during the sensitive adolescent period.

## Supporting information


**Table S1.** Demographic information across all timepoints.
**Figure S1.** PoP‐ORL model parameter recovery at each timepoint.
**Figure S2.** Associations between PoP‐ORL recovered parameters – reward learning rate and win/loss frequency sensitivity – at each timepoint.
**Figure S3.** PoP‐ORL model posterior predictive checks at each timepoint.
**Table S2.** Test–retest reliability for the traditional and computational performance metrics.
**Figure S4.** Increased punishment learning rate and win/loss frequency sensitivity support overall good performance.
**Table S3.** Nested model results for traditional summary scores and PoP‐ORL parameter means.

## Data Availability

Data and code will be made available upon publication at the following link: https://osf.io/yh458/wiki/PoP%20IGT%20adolescent%20trajectories/.
