# Supplementary material for: Reward‐specific learning parameters change across normative adolescent development and are blunted in youth with high risk for depression
Source: J Child Psychol Psychiatry. 2025 Dec 22;67(6):963–75. doi: 10.1111/jcpp.70086 (PMC13047750; doi:10.1111/jcpp.70086)
Supplement: Supplementary file 1 — Table S1. Demographic information across all timepoints. Figure S1. PoP‐ORL model parameter recovery at each timepoint. Figure S2. Associations between PoP‐ORL recovered parameters – reward learning rate and win/loss frequency sensitivity – at each timepoint. Figure S3. PoP‐ORL model posterior predictive checks at each timepoint. Table S2. Test–retest reliability for the traditional and computational performance metrics. Figure S4. Increased punishment learning rate and win/loss frequency sensitivity support overall good performance. Table S3. Nested model results for traditional summary scores and PoP‐ORL parameter means. [file JCPP-67-963-s001.docx]

**Reward-specific learning parameters change across normative adolescent development and are blunted in youth with high risk for depression.**

**Supporting Information**

**Table S1. Demographic information across all timepoints.**

**Figure S1. PoP-ORL model parameter recovery at each timepoint.** Parameter recovery was generally in the good-to-excellent range across the timepoints for all parameters except for *Arew* where parameter recovery correlations were in the range of .59-.67

**Figure S2. Associations between PoP-ORL recovered parameters—reward learning rate and win/loss frequency sensitivity—at each timepoint.** The reward-specific recovered parameters, *Arew* and *βf*, showed weak or no correlation with one another, suggesting independence of these parameters and their respective developmental trajectories.

**

**

**Figure S3. PoP-ORL model posterior predictive checks at each timepoint.** Posterior predicative checks demonstrated that the model-simulated data was a good fit to observed data.

**Table S2. Test-retest reliability for the traditional and computational performance metrics.**

| **Learning Metric** | **Timepoint 1** | **Timepoint 2** | ***r*** | ***p*** |
| --- | --- | --- | --- | --- |
| Net Proportion Played | T1 | T2 | .43 | < .001 |
| Net Proportion Played | T2 | T3 | .40 | < .001 |
| Net Proportion Played | T3 | T4 | .40 | < .001 |
| Net Proportion Played | T4 | T5 | .73 | < .001 |
| Good Deck Proportion Played | T1 | T2 | .41 | < .001 |
| Good Deck Proportion Played | T2 | T3 | .36 | < .001 |
| Good Deck Proportion Played | T3 | T4 | .46 | < .001 |
| Good Deck Proportion Played | T4 | T5 | .75 | < .001 |
| Bad Deck Proportion Played | T1 | T2 | .43 | < .001 |
| Bad Deck Proportion Played | T2 | T3 | .50 | < .001 |
| Bad Deck Proportion Played | T3 | T4 | .54 | < .001 |
| Bad Deck Proportion Played | T4 | T5 | .69 | < .001 |
| Reward Learning Rate (Arew) | T1 | T2 | .22 | .005 |
| Reward Learning Rate (Arew) | T2 | T3 | .11 | .249 |
| Reward Learning Rate (Arew) | T3 | T4 | .13 | .248 |
| Reward Learning Rate (Arew) | T4 | T5 | .36 | .009 |
| Punishment Learning Rate (Apun) | T1 | T2 | .38 | < .001 |
| Punishment Learning Rate (Apun) | T2 | T3 | .29 | .001 |
| Punishment Learning Rate (Apun) | T3 | T4 | .48 | < .001 |
| Punishment Learning Rate (Apun) | T4 | T5 | .58 | < .001 |
| Win/Loss Frequency Effect (BetaF) | T1 | T2 | .41 | < .001 |
| Win/Loss Frequency Effect (BetaF) | T2 | T3 | .25 | .006 |
| Win/Loss Frequency Effect (BetaF) | T3 | T4 | .37 | < .001 |
| Win/Loss Frequency Effect (BetaF) | T4 | T5 | .55 | < .001 |
| Go Bias (BetaB) | T1 | T2 | .31 | < .001 |
| Go Bias (BetaB) | T2 | T3 | .41 | < .001 |
| Go Bias (BetaB) | T3 | T4 | .43 | < .001 |
| Go Bias (BetaB) | T4 | T5 | .51 | < .001 |

**Figure S4.** **Increased punishment learning rate and win/loss frequency sensitivity support overall good performance.** PoP-ORL parameter means are plotted against ‘net proportion play’ summary scores at each timepoint to illustrate how learning parameters impact overall good performance across task administrations. Whole-sample fit lines representing Pearson’s correlations are overlaid in purple. Data points from youth with a maternal history of depression (no maternal history of depression) are plotted in orange (green), with respective orange and green sub-group fit lines underlaid. Punishment learning rate and win/loss frequency sensitivity appear to support overall good net performance across time, while reward learning rate and go bias appear to support good performance at later task administrations.

**
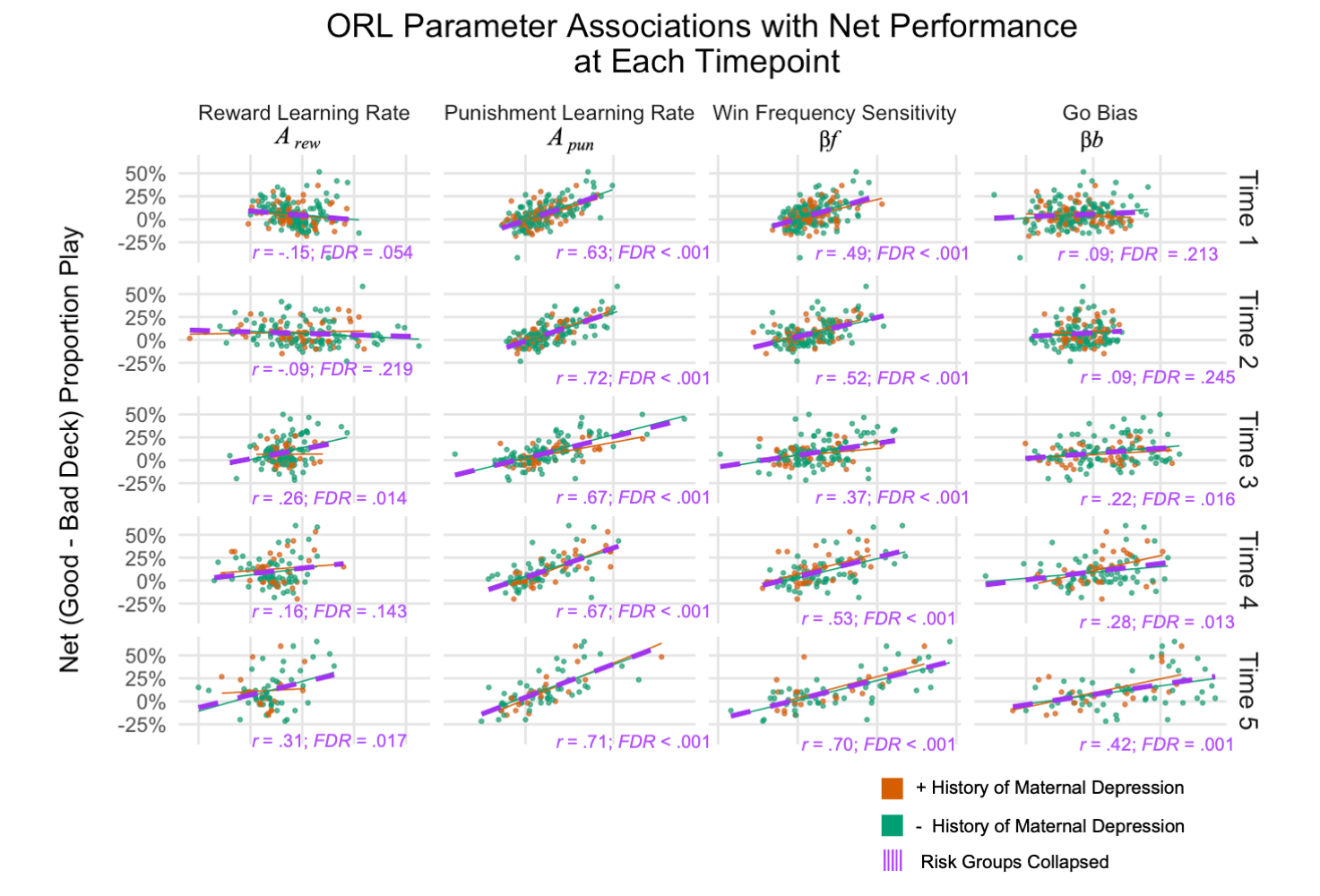
**

**Table S3. Nested model results for traditional summary scores and PoP-ORL parameter means.** Reported coefficients are unstandardized. Model 1 examines unconditional linear change in metrics across age. Model 2 examines unconditional nonlinear change in metrics across age. Model 3 examines maternal history of depression as a predictor of behavioral metric growth trajectories, building on the winning unconditional model (linear or quadratic). Model 4, the full model, adds covariates including child sex, K-BIT nonverbal IQ, and task administration modality (in-lab/E-prime vs. remote/Pavlovia) to the conditional growth models.

| Traditional Scoring: Net Proportion Played (Good Deck – Bad Deck) | | | | | |
| --- | --- | --- | --- | --- | --- |
|  |  | Model | | | |
|  |  | 1 | 2 | 3 | 4 |
| **Fixed Effects** |  |  |  |  |  |
| Constant |  | .05 ^***^ [.04 – .07] | .05 ^***^ [.04 – .07] | .05 ^***^ [.04 – .07] | .08 ^***^ [.03 – .12] |
| Age |  | .02 ^***^ [.01 – .02] | .02^**^ [.01 – .03] | .01 ^**^ [.01 – .02] | .01 ^**^ [.00 – .02] |
| Age^2^ |  |  | .00 [-.00 – .00] |  |  |
| Maternal Hx Depression |  |  |  | -.00 [-.03 – .03] | -.01 [-.04 – .01] |
| Age * Maternal Hx Depression |  |  |  | .01 [-.01 – .02] | .01 [-.01 – .02] |
| K-BIT Nonverbal Standard IQ |  |  |  |  | .00 ^***^ [.00 – .00] |
| Child Sex |  |  |  |  | .00 [-.03 – .03] |
| On-site Administration |  |  |  |  | -.02 [-.06 – .02] |
| **Random Effects** |  |  |  |  |  |
| σ^2^ |  | .01 | .01 | .01 | .01 |
| τ_00_ _id_ |  | .00 | .00 | .00 | .00 |
| τ_11_ _id.age_ |  | .00 | .00 | .00 | .00 |
| N _id_ |  | 208 | 208 | 208 | 208 |
| **Fit Statistics** |  |  |  |  |  |
| AIC |  | -846.5 | -844.6 | -843.0 | -875.5 |
| BIC |  | -823.9 | -817.5 | -811.4 | -830.4 |
| Loglik |  | 428.2 | 428.3 | 428.5 | 447.7 |
| Deviance |  | -856.5 | -856.6 | -857.0 | -895.5 |
| **Model Comparisons** |  |  |  |  |  |
| compared to Model 1 |  |  | $X_{1}^{2}$ = .07 | $X_{2}^{2}$ = .52 | $X_{5}^{2}$ = 38.99 ^***^ |
| compared to Model 2 |  |  |  |  |  |
| compared to Model 3 |  |  |  |  | $X_{3}^{2}$ = 38.47 ^***^ |
| ICC |  | .29 | .29 | .28 | .21 |
| Pseudo R^2^ (total) |  | .33 | .33 | .33 | .34 |
| * p < .05  ** p < .01  *** p < .001 |  |  |  |  |  |
|  |  |  |  |  |  |
| Traditional Scoring: Good Deck Proportion Played | | | | | |
|  |  | Model | | | |
|  |  | 1 | 2 | 3 | 4 |
| **Fixed Effects** |  |  |  |  |  |
| Constant |  | .73 ^***^ [.72 – .75] | .73 ^***^ [.72 – .75] | .74 ^***^ [.72 – .76] | .80 ^***^ [.75 – .85] |
| Age |  | .01 ^*^ [.00 – .02] | .01 [-.00 – .02] | .01 [-.00 – .02] | .00  [-.01 – .01] |
| Age^2^ |  |  | .00 [-.00 – .00] |  |  |
| Maternal Hx Depression |  |  |  | -.02 [-.06 – .01] | -.03 [-.06 – .00] |
| Age * Maternal Hx Depression |  |  |  | .01 [-.01 – .03] | .01 [-.01 – .03] |
| K-BIT Nonverbal Standard IQ |  |  |  |  | .00 ^**^ [.00 – .00] |
| Child Sex |  |  |  |  | -.03 [-.06 – .01] |
| On-site Administration |  |  |  |  | -.04 [-.08 – .00] |
| **Random Effects** |  |  |  |  |  |
| σ^2^ |  | .01 | .01 | .01 | .01 |
| τ_00_ _id_ |  | .01 | .01 | .01 | .01 |
| τ_11_ _id.age_ |  | .00 | .00 | .00 | .00 |
| N _id_ |  | 208 | 208 | 208 | 208 |
| **Fit Statistics** |  |  |  |  |  |
| AIC |  | -757.5 | -755.6 | -756.0 | -760.9 |
| BIC |  | -735.0 | -728.5 | -724.5 | -715.9 |
| Loglik |  | 383.8 | 383.8 | 385.0 | 390.5 |
| Deviance |  | -767.5 | -767.6 | -770.0 | -780.9 |
| **Model Comparisons** |  |  |  |  |  |
| compared to Model 1 |  |  | $X_{1}^{2}$ = .05 | $X_{2}^{2}$ = 2.50 | $X_{5}^{2}$ = 13.42 ^*^ |
| compared to Model 2 |  |  |  |  |  |
| compared to Model 3 |  |  |  |  | $X_{3}^{2}$ = 10.92 ^*^ |
| ICC |  | .36 | .36 | .35 | .32 |
| Pseudo R^2^ (total) |  | .37 | .37 | .36 | .36 |
| * p < .05  ** p < .01  *** p < .001 |  |  |  |  |  |

| Traditional Scoring: Bad Deck Proportion Played | | | | | |
| --- | --- | --- | --- | --- | --- |
|  |  | Model | | | |
|  |  | 1 | 2 | 3 | 4 |
| **Fixed Effects** |  |  |  |  |  |
| Constant |  | .68 ^***^ [.66 – .70] | .68 ^***^ [.66 – .70] | .69 ^***^ [.67 – .71] | .72 ^***^ [.67 – .77] |
| Age |  | -.01 [-.01 – .00] | -.01 [-.02 – .00] | -.01 [-.02 – .00] | -.01 [-.02 – .00] |
| Age^2^ |  |  | -.00 [-.00 – .00] |  |  |
| Maternal Hx Depression |  |  |  | -.02 [-.06 – .01] | -.02 [-.05 – .02] |
| Age * Maternal Hx Depression |  |  |  | .00 [-.01 – .02] | .00 [-.01 – .02] |
| K-BIT Nonverbal Standard IQ |  |  |  |  | -.00 ^**^ [-.00 – -.00] |
| Child Sex |  |  |  |  | -.03 [-.06 – .00] |
| On-site Administration |  |  |  |  | -.01 [-.05 – .03] |
| **Random Effects** |  |  |  |  |  |
| σ^2^ |  | .01 | .01 | .01 | .01 |
| τ_00_ _id_ |  | .01 | .01 | .01 | .01 |
| τ_11_ _id.age_ |  | .00 | .00 | .00 | .00 |
| N _id_ |  | 208 | 208 | 208 | 208 |
| **Fit Statistics** |  |  |  |  |  |
| AIC |  | -752.6 | -750.7 | -750.0 | -755.3 |
| BIC |  | -730.1 | -723.6 | -718.4 | -710.2 |
| Loglik |  | 381.3 | 381.3 | 382.0 | 387.6 |
| Deviance |  | -762.6 | -762.7 | -764.0 | -775.3 |
| **Model Comparisons** |  |  |  |  |  |
| compared to Model 1 |  |  | $X_{1}^{2}$ = .02 | $X_{2}^{2}$ = 1.33 | $X_{5}^{2}$ = 12.63 ^*^ |
| compared to Model 2 |  |  |  |  |  |
| compared to Model 3 |  |  |  |  | $X_{3}^{2}$ = 11.30 ^*^ |
| ICC |  | .39 | .39 | .39 | .36 |
| Pseudo R^2^ (total) |  | .40 | .40 | .39 | .39 |
| * p < .05  ** p < .01  *** p < .001 |  |  |  |  |  |

| Computational Modeling: Reward Learning Rate (Arew) | | | | | |
| --- | --- | --- | --- | --- | --- |
|  |  | Model | | | |
|  |  | 1 | 2 | 3 | 4 |
| **Fixed Effects** |  |  |  |  |  |
| Constant |  | -1.57 ^***^ [-1.60 – -1.55] | -1.57 ^***^ [-1.60 – -1.55] | -1.55 ^***^ [-1.59 – -1.52] | -1.67 ^***^ [-1.76 – -1.59] |
| Age |  | -.04 ^***^ [-.05 – -.03] | -.04 ^***^ [-.06 – -.02] | -.05 ^***^ [-.06 – -.03] | -.04 ^***^ [-.06 – -.03] |
| Age^2^ |  |  | -.00 [-.01 – .00] |  |  |
| Maternal Hx Depression |  |  |  | -.06 ^*^ [-.11 – -.00] | -.06 ^*^ [-.11 – -.00] |
| Age * Maternal Hx Depression |  |  |  | .02 [-.00 – .05] | .02 [-.00 – .05] |
| K-BIT Nonverbal Standard IQ |  |  |  |  | .00 [-.00 – .00] |
| Child Sex |  |  |  |  | .00 [-.04 – .05] |
| On-site Administration |  |  |  |  | .12 ^**^ [.04 – .20] |
| **Random Effects** |  |  |  |  |  |
| σ^2^ |  | .06 | .06 | .06 | .06 |
| τ_00_ _id_ |  | .01 | .01 | .01 | .01 |
| τ_11_ _id.age_ |  | .00 | .00 | .00 | .00 |
| N _id_ |  | 208 | 208 | 208 | 208 |
| **Fit Statistics** |  |  |  |  |  |
| AIC |  | 131.9 | 133.8 | 130.9 | 126.2 |
| BIC |  | 154.4 | 160.8 | 162.5 | 171.3 |
| Loglik |  | -60.9 | -60.9 | -58.5 | -53.1 |
| Deviance |  | 121.9 | 121.8 | 116.9 | 106.2 |
| **Model Comparisons** |  |  |  |  |  |
| compared to Model 1 |  |  | $X_{1}^{2}$ = .12 | $X_{2}^{2}$ = 4.98 | $X_{5}^{2}$ = 15.65 ^**^ |
| compared to Model 2 |  |  |  |  |  |
| compared to Model 3 |  |  |  |  | $X_{3}^{2}$ = 10.66 ^*^ |
| ICC |  | .13 | .13 | .12 | .12 |
| Pseudo R^2^ (total) |  | .20 | .20 | .20 | .20 |
| * p < .05  ** p < .01  *** p < .001 |  |  |  |  |  |

| Computational Modeling: Punishment Learning Rate (Apun) | | | | | |
| --- | --- | --- | --- | --- | --- |
|  |  | Model | | | |
|  |  | 1 | 2 | 3 | 4 |
| **Fixed Effects** |  |  |  |  |  |
| Constant |  | -1.80 ^***^ [-1.83 – -1.77] | -1.81 ^***^ [-1.84 – -1.78] | -1.80 ^***^ [-1.84 – -1.76] | -1.81 ^***^ [-1.91 – -1.71] |
| Age |  | .02 [-.00 – .03] | .01 [-.02 – .03] | .01 [-.01 – .03] | .01 [-.01 – .03] |
| Age^2^ |  |  | .00 [-.00 – .01] |  |  |
| Maternal Hx Depression |  |  |  | -.00 [-.07 – .06] | -.02 [-.08 – .04] |
| Age * Maternal Hx Depression |  |  |  | .02 [-.01 – .06] | .02 [-.01 – .06] |
| K-BIT Nonverbal Standard IQ |  |  |  |  | .01 ^***^ [.00 – .01] |
| Child Sex |  |  |  |  | .01 [-.05 – .07] |
| On-site Administration |  |  |  |  | .01 [-.08 – .09] |
| **Random Effects** |  |  |  |  |  |
| σ^2^ |  | .06 | .06 | .06 | .06 |
| τ_00_ _id_ |  | .02 | .02 | .02 | .01 |
| τ_11_ _id.age_ |  | .00 | .00 | .00 | .00 |
| N _id_ |  | 208 | 208 | 208 | 208 |
| **Fit Statistics** |  |  |  |  |  |
| AIC |  | 248.2 | 248.4 | 250.6 | 226.9 |
| BIC |  | 270.7 | 275.5 | 282.2 | 272.0 |
| Loglik |  | -119.1 | -118.2 | -118.3 | -103.5 |
| Deviance |  | 238.2 | 236.4 | 236.6 | 206.9 |
| **Model Comparisons** |  |  |  |  |  |
| compared to Model 1 |  |  | $X_{1}^{2}$ = 1.75 | $X_{2}^{2}$ = 1.53 | $X_{5}^{2}$ = 31.23 ^***^ |
| compared to Model 2 |  |  |  |  |  |
| compared to Model 3 |  |  |  |  | $X_{3}^{2}$ = 29.70 ^***^ |
| ICC |  | .20 | .21 | .20 | .15 |
| Pseudo R^2^ (total) |  | .21 | .22 | .22 | .24 |
| * p < .05  ** p < .01  *** p < .001 |  |  |  |  |  |

| Computational Modeling: Win Frequency Effect (BetaF) | | | | | |
| --- | --- | --- | --- | --- | --- |
|  |  | Model | | | |
|  |  | 1 | 2 | 3 | 4 |
| **Fixed Effects** |  |  |  |  |  |
| Constant |  | 1.71 ^***^ [1.36 – 2.06] | 1.64 ^***^ [1.29 – 2.00] | 1.82 ^***^ [1.38 – 2.25] | 4.42 ^***^ [3.25 – 5.59] |
| Age |  | .47 ^***^ [.26 – .68] | .31 ^*^ [.04 – .58] | .46 ^***^ [.20 – .72] | .31 ^*^ [.06 – .56] |
| Age^2^ |  |  | .08 [-.00 – .15] |  |  |
| Maternal Hx Depression |  |  |  | -.29 [-1.02 – .44] | -.52 [-1.21 – .17] |
| Age * Maternal Hx Depression |  |  |  | .01 [-.45 – .46] | .04 [-.39 – .47] |
| K-BIT Nonverbal Standard IQ |  |  |  |  | .05 ^***^ [.03 – .07] |
| Child Sex |  |  |  |  | -.10 [-.75 – .56] |
| On-site Administration |  |  |  |  | -2.50 ^***^ [-3.51 – -1.48] |
| **Random Effects** |  |  |  |  |  |
| σ^2^ |  | 8.26 | 8.18 | 8.27 | 8.15 |
| τ_00_ _id_ |  | 1.73 | 1.74 | 1.70 | 1.21 |
| τ_11_ _id.age_ |  | .92 | .94 | .92 | .75 |
| N _id_ |  | 208 | 208 | 208 | 208 |
| **Fit Statistics** |  |  |  |  |  |
| AIC |  | 3564.3 | 3562.8 | 3567.6 | 3531.3 |
| BIC |  | 3586.8 | 3589.9 | 3599.2 | 3576.4 |
| Loglik |  | -1777.1 | -1775.4 | -1776.8 | -1755.6 |
| Deviance |  | 3554.3 | 3550.8 | 3553.6 | 3511.3 |
| **Model Comparisons** |  |  |  |  |  |
| compared to Model 1 |  |  | $X_{1}^{2}$ = 3.41 | $X_{2}^{2}$ = .62 | $X_{5}^{2}$ = 42.99 ^***^ |
| compared to Model 2 |  |  |  |  |  |
| compared to Model 3 |  |  |  |  | $X_{3}^{2}$ = 42.36 ^***^ |
| ICC |  | .17 | .18 | .17 | .13 |
| Pseudo R^2^ (total) |  | .23 | .25 | .23 | .26 |
| * p < .05  ** p < .01  *** p < .001 |  |  |  |  |  |

| Computational Modeling: Go Bias (BetaB) | | | | | |
| --- | --- | --- | --- | --- | --- |
|  |  | Model | | | |
|  |  | 1 | 2 | 3 | 4 |
| **Fixed Effects** |  |  |  |  |  |
| Constant |  | .86 ^***^ [.81 – .92] | .84 ^***^ [.79 – .90] | .87 ^***^ [.80 – .94] | 1.25 ^***^ [1.08 – 1.43] |
| Age |  | .09 ^***^ [.07 – .12] | .05 ^*^ [.01 – .08] | .05 ^*^ [.00 – .10] | .03 [-.01 – .08] |
| Age^2^ |  |  | .02 ^***^ [.01 – .03] | .02 ^**^ [.01 – .03] | .01 ^*^ [-.00 – .03] |
| Maternal Hx Depression |  |  |  | -.07 [-.18 – .05] | -.08 [-.20 – .03] |
| Age * Maternal Hx Depression |  |  |  | -.01 [-.09 – .07] | -.01 [-.09 – .07] |
| Age^2^ * Maternal Hx Depression |  |  |  | .00 [-.02 – .02] | .00 [-.02 – .03] |
| K-BIT Nonverbal Standard IQ |  |  |  |  | .00  [.00 – .01] |
| Child Sex |  |  |  |  | -.06 [-.16 – .04] |
| On-site Administration |  |  |  |  | -.33 ^***^ [-.48 – -.19] |
| **Random Effects** |  |  |  |  |  |
| σ^2^ |  | .17 | .16 | .16 | .17 |
| τ_00_ _id_ |  | .07 | .06 | .06 | .05 |
| τ_11_ _id.age_ |  | .01 | .01 | .01 | .01 |
| N _id_ |  | 208 | 208 | 208 | 208 |
| **Fit Statistics** |  |  |  |  |  |
| AIC |  | 954.4 | 944.1 | 948.5 | 933.3 |
| BIC |  | 977.0 | 971.2 | 989.1 | 987.4 |
| Loglik |  | -472.2 | -466.1 | -465.3 | -454.6 |
| Deviance |  | 944.4 | 932.1 | 930.5 | 909.3 |
| **Model Comparisons** |  |  |  |  |  |
| compared to Model 1 |  |  | $X_{1}^{2}$ = 12.26 ^***^ |  |  |
| compared to Model 2 |  |  |  | $X_{4}^{2}$ = 1.61 | $X_{6}^{2}$ = 22.88 ^***^ |
| compared to Model 3 |  |  |  |  | $X_{3}^{2}$ = 21.27 ^***^ |
| ICC |  | .29 | .28 | .27 | .22 |
| Pseudo R^2^ (total) |  | .37 | .40 | .39 | .35 |
| * p < .05  ** p < .01  *** p < .001 |  |  |  |  |  |
