# Supplementary material for: Reward-specific learning parameters change across normative adolescent development and are blunted in youth with high risk for depression
Source: J Child Psychol Psychiatry. Author manuscript; Available in PMC 2026 Apr 4. (PMC13047750; doi:10.1111/jcpp.70086)
Supplement: Supplementary file 1 [file NIHMS2144980-supplement-Supplementary_Material.docx]

**Supplemental Table 1. Demographic information across all timepoints.** 

**Supplemental Figure 1. PoP-ORL model parameter recovery at each timepoint.** Parameter recovery was generally in the good-to-excellent range across the timepoints for all parameters except for *Arew* where parameter recovery correlations were in the range of .59-.67

**Supplemental Figure 2. Associations between PoP-ORL recovered parameters—reward learning rate and win/loss frequency sensitivity—at each timepoint.** The reward-specific recovered parameters, *Arew* and *βf*, showed weak or no correlation with one another, suggesting independence of these parameters and their respective developmental trajectories.	

**Supplemental Figure 3. PoP-ORL model posterior predictive checks at each timepoint.** Posterior predicative checks demonstrated that the model-simulated data was a good fit to observed data. 

**Supplemental Table 2. Test-retest reliability for the traditional and computational performance metrics.** 

| **Learning Metric** | **Timepoint 1** | **Timepoint 2** | ***r*** | ***p*** |
| --- | --- | --- | --- | --- |
| Net Proportion Played | T1 | T2 | .43 | < .001 |
| Net Proportion Played | T2 | T3 | .40 | < .001 |
| Net Proportion Played | T3 | T4 | .40 | < .001 |
| Net Proportion Played | T4 | T5 | .73 | < .001 |
| Good Deck Proportion Played | T1 | T2 | .41 | < .001 |
| Good Deck Proportion Played | T2 | T3 | .36 | < .001 |
| Good Deck Proportion Played | T3 | T4 | .46 | < .001 |
| Good Deck Proportion Played | T4 | T5 | .75 | < .001 |
| Bad Deck Proportion Played | T1 | T2 | .43 | < .001 |
| Bad Deck Proportion Played | T2 | T3 | .50 | < .001 |
| Bad Deck Proportion Played | T3 | T4 | .54 | < .001 |
| Bad Deck Proportion Played | T4 | T5 | .69 | < .001 |
| Reward Learning Rate (Arew) | T1 | T2 | .22 | .005 |
| Reward Learning Rate (Arew) | T2 | T3 | .11 | .249 |
| Reward Learning Rate (Arew) | T3 | T4 | .13 | .248 |
| Reward Learning Rate (Arew) | T4 | T5 | .36 | .009 |
| Punishment Learning Rate (Apun) | T1 | T2 | .38 | < .001 |
| Punishment Learning Rate (Apun) | T2 | T3 | .29 | .001 |
| Punishment Learning Rate (Apun) | T3 | T4 | .48 | < .001 |
| Punishment Learning Rate (Apun) | T4 | T5 | .58 | < .001 |
| Win/Loss Frequency Effect (BetaF) | T1 | T2 | .41 | < .001 |
| Win/Loss Frequency Effect (BetaF) | T2 | T3 | .25 | .006 |
| Win/Loss Frequency Effect (BetaF) | T3 | T4 | .37 | < .001 |
| Win/Loss Frequency Effect (BetaF) | T4 | T5 | .55 | < .001 |
| Go Bias (BetaB) | T1 | T2 | .31 | < .001 |
| Go Bias (BetaB) | T2 | T3 | .41 | < .001 |
| Go Bias (BetaB) | T3 | T4 | .43 | < .001 |
| Go Bias (BetaB) | T4 | T5 | .51 | < .001 |

**Supplemental Figure 4.** **Increased punishment learning rate and win/loss frequency sensitivity support overall good performance.** PoP-ORL parameter means are plotted against ‘net proportion play’ summary scores at each timepoint to illustrate how learning parameters impact overall good performance across task administrations. Whole-sample fit lines representing Pearson’s correlations are overlaid in purple. Data points from youth with a maternal history of depression (no maternal history of depression) are plotted in orange (green), with respective orange and green sub-group fit lines underlaid. Punishment learning rate and win/loss frequency sensitivity appear to support overall good net performance across time, while reward learning rate and go bias appear to support good performance at later task administrations. 

**Supplemental Table 3. Nested model results for traditional summary scores and PoP-ORL parameter means.** Reported coefficients are unstandardized. Model 1 examines unconditional linear change in metrics across age. Model 2 examines unconditional nonlinear change in metrics across age. Model 3 examines maternal history of depression as a predictor of behavioral metric growth trajectories, building on the winning unconditional model (linear or quadratic). Model 4, the full model, adds covariates including child sex, K-BIT nonverbal IQ, and task administration modality (in-lab/E-prime vs. remote/Pavlovia) to the conditional growth models. 

	

| Traditional Scoring:  Net Proportion Played (Good Deck – Bad Deck) |  |  |  |  |  |
| --- | --- | --- | --- | --- | --- |
|  |  | Model |  |  |  |
|  |  | 1 | 2 | 3 | 4 |
| **Fixed Effects** |  |  |  |  |  |
| Constant |  | .05 ^***^ [.04 – .07] | .05 ^***^ [.04 – .07] | .05 ^***^ [.04 – .07] | .08 ^***^ [.03 – .12] |
| Age |  | .02 ^***^ [.01 – .02] | .02^**^ [.01 – .03] | .01 ^**^ [.01 – .02] | .01 ^**^ [.00 – .02] |
| Age^2^ |  |  | .00 [-.00 – .00] |  |  |
| Maternal Hx Depression |  |  |  | -.00 [-.03 – .03] | -.01 [-.04 – .01] |
| Age * Maternal Hx Depression |  |  |  | .01 [-.01 – .02] | .01 [-.01 – .02] |
| K-BIT Nonverbal Standard IQ |  |  |  |  | .00 ^***^ [.00 – .00] |
| Child Sex |  |  |  |  | .00 [-.03 – .03] |
| On-site Administration |  |  |  |  | -.02 [-.06 – .02] |
| **Random Effects** |  |  |  |  |  |
| σ^2^ |  | .01 | .01 | .01 | .01 |
| τ_00_ _id_ |  | .00 | .00 | .00 | .00 |
| τ_11_ _id.age_ |  | .00 | .00 | .00 | .00 |
| N _id_ |  | 208 | 208 | 208 | 208 |
| **Fit Statistics** |  |  |  |  |  |
| AIC |  | -846.5 | -844.6 | -843.0 | -875.5 |
| BIC |  | -823.9 | -817.5 | -811.4 | -830.4 |
| Loglik |  | 428.2 | 428.3 | 428.5 | 447.7 |
| Deviance |  | -856.5 | -856.6 | -857.0 | -895.5 |
| **Model Comparisons** |  |  |  |  |  |
| compared to Model 1 |  |  | $X_{1}^{2}$ = .07 | $X_{2}^{2}$ = .52 | $X_{5}^{2}$ = 38.99 ^***^ |
| compared to Model 2 |  |  |  |  |  |
| compared to Model 3 |  |  |  |  | $X_{3}^{2}$ = 38.47 ^***^ |
| ICC |  | .29 | .29 | .28 | .21 |
| Pseudo R^2^ (total) |  | .33 | .33 | .33 | .34 |
| * p < .05   ** p < .01 *** p < .001 |  |  |  |  |  |
|  |  |  |  |  |  |
| Traditional Scoring:  Good Deck Proportion Played |  |  |  |  |  |
|  |  | Model |  |  |  |
|  |  | 1 | 2 | 3 | 4 |
| **Fixed Effects** |  |  |  |  |  |
| Constant |  | .73 ^***^ [.72 – .75] | .73 ^***^ [.72 – .75] | .74 ^***^ [.72 – .76] | .80 ^***^ [.75 – .85] |
| Age |  | .01 ^*^ [.00 – .02] | .01 [-.00 – .02] | .01 [-.00 – .02] | .00  [-.01 – .01] |
| Age^2^ |  |  | .00 [-.00 – .00] |  |  |
| Maternal Hx Depression |  |  |  | -.02 [-.06 – .01] | -.03 [-.06 – .00] |
| Age * Maternal Hx Depression |  |  |  | .01 [-.01 – .03] | .01 [-.01 – .03] |
| K-BIT Nonverbal Standard IQ |  |  |  |  | .00 ^**^ [.00 – .00] |
| Child Sex |  |  |  |  | -.03 [-.06 – .01] |
| On-site Administration |  |  |  |  | -.04 [-.08 – .00] |
| **Random Effects** |  |  |  |  |  |
| σ^2^ |  | .01 | .01 | .01 | .01 |
| τ_00_ _id_ |  | .01 | .01 | .01 | .01 |
| τ_11_ _id.age_ |  | .00 | .00 | .00 | .00 |
| N _id_ |  | 208 | 208 | 208 | 208 |
| **Fit Statistics** |  |  |  |  |  |
| AIC |  | -757.5 | -755.6 | -756.0 | -760.9 |
| BIC |  | -735.0 | -728.5 | -724.5 | -715.9 |
| Loglik |  | 383.8 | 383.8 | 385.0 | 390.5 |
| Deviance |  | -767.5 | -767.6 | -770.0 | -780.9 |
| **Model Comparisons** |  |  |  |  |  |
| compared to Model 1 |  |  | $X_{1}^{2}$ = .05 | $X_{2}^{2}$ = 2.50 | $X_{5}^{2}$ = 13.42 ^*^ |
| compared to Model 2 |  |  |  |  |  |
| compared to Model 3 |  |  |  |  | $X_{3}^{2}$ = 10.92 ^*^ |
| ICC |  | .36 | .36 | .35 | .32 |
| Pseudo R^2^ (total) |  | .37 | .37 | .36 | .36 |
| * p < .05   ** p < .01 *** p < .001 |  |  |  |  |  |

| Traditional Scoring:  Bad Deck Proportion Played |  |  |  |  |  |
| --- | --- | --- | --- | --- | --- |
|  |  | Model |  |  |  |
|  |  | 1 | 2 | 3 | 4 |
| **Fixed Effects** |  |  |  |  |  |
| Constant |  | .68 ^***^ [.66 – .70] | .68 ^***^ [.66 – .70] | .69 ^***^ [.67 – .71] | .72 ^***^ [.67 – .77] |
| Age |  | -.01 [-.01 – .00] | -.01 [-.02 – .00] | -.01 [-.02 – .00] | -.01 [-.02 – .00] |
| Age^2^ |  |  | -.00 [-.00 – .00] |  |  |
| Maternal Hx Depression |  |  |  | -.02 [-.06 – .01] | -.02 [-.05 – .02] |
| Age * Maternal Hx Depression |  |  |  | .00 [-.01 – .02] | .00 [-.01 – .02] |
| K-BIT Nonverbal Standard IQ |  |  |  |  | -.00 ^**^ [-.00 – -.00] |
| Child Sex |  |  |  |  | -.03 [-.06 – .00] |
| On-site Administration |  |  |  |  | -.01 [-.05 – .03] |
| **Random Effects** |  |  |  |  |  |
| σ^2^ |  | .01 | .01 | .01 | .01 |
| τ_00_ _id_ |  | .01 | .01 | .01 | .01 |
| τ_11_ _id.age_ |  | .00 | .00 | .00 | .00 |
| N _id_ |  | 208 | 208 | 208 | 208 |
| **Fit Statistics** |  |  |  |  |  |
| AIC |  | -752.6 | -750.7 | -750.0 | -755.3 |
| BIC |  | -730.1 | -723.6 | -718.4 | -710.2 |
| Loglik |  | 381.3 | 381.3 | 382.0 | 387.6 |
| Deviance |  | -762.6 | -762.7 | -764.0 | -775.3 |
| **Model Comparisons** |  |  |  |  |  |
| compared to Model 1 |  |  | $X_{1}^{2}$ = .02 | $X_{2}^{2}$ = 1.33 | $X_{5}^{2}$ = 12.63 ^*^ |
| compared to Model 2 |  |  |  |  |  |
| compared to Model 3 |  |  |  |  | $X_{3}^{2}$ = 11.30 ^*^ |
| ICC |  | .39 | .39 | .39 | .36 |
| Pseudo R^2^ (total) |  | .40 | .40 | .39 | .39 |
| * p < .05   ** p < .01 *** p < .001 |  |  |  |  |  |

| Computational Modeling:  Reward Learning Rate (Arew) |  |  |  |  |  |
| --- | --- | --- | --- | --- | --- |
|  |  | Model |  |  |  |
|  |  | 1 | 2 | 3 | 4 |
| **Fixed Effects** |  |  |  |  |  |
| Constant |  | -1.57 ^***^ [-1.60 – -1.55] | -1.57 ^***^ [-1.60 – -1.55] | -1.55 ^***^ [-1.59 – -1.52] | -1.67 ^***^ [-1.76 – -1.59] |
| Age |  | -.04 ^***^ [-.05 – -.03] | -.04 ^***^ [-.06 – -.02] | -.05 ^***^ [-.06 – -.03] | -.04 ^***^ [-.06 – -.03] |
| Age^2^ |  |  | -.00 [-.01 – .00] |  |  |
| Maternal Hx Depression |  |  |  | -.06 ^*^ [-.11 – -.00] | -.06 ^*^ [-.11 – -.00] |
| Age * Maternal Hx Depression |  |  |  | .02 [-.00 – .05] | .02 [-.00 – .05] |
| K-BIT Nonverbal Standard IQ |  |  |  |  | .00 [-.00 – .00] |
| Child Sex |  |  |  |  | .00 [-.04 – .05] |
| On-site Administration |  |  |  |  | .12 ^**^ [.04 – .20] |
| **Random Effects** |  |  |  |  |  |
| σ^2^ |  | .06 | .06 | .06 | .06 |
| τ_00_ _id_ |  | .01 | .01 | .01 | .01 |
| τ_11_ _id.age_ |  | .00 | .00 | .00 | .00 |
| N _id_ |  | 208 | 208 | 208 | 208 |
| **Fit Statistics** |  |  |  |  |  |
| AIC |  | 131.9 | 133.8 | 130.9 | 126.2 |
| BIC |  | 154.4 | 160.8 | 162.5 | 171.3 |
| Loglik |  | -60.9 | -60.9 | -58.5 | -53.1 |
| Deviance |  | 121.9 | 121.8 | 116.9 | 106.2 |
| **Model Comparisons** |  |  |  |  |  |
| compared to Model 1 |  |  | $X_{1}^{2}$ = .12 | $X_{2}^{2}$ = 4.98 | $X_{5}^{2}$ = 15.65 ^**^ |
| compared to Model 2 |  |  |  |  |  |
| compared to Model 3 |  |  |  |  | $X_{3}^{2}$ = 10.66 ^*^ |
| ICC |  | .13 | .13 | .12 | .12 |
| Pseudo R^2^ (total) |  | .20 | .20 | .20 | .20 |
| * p < .05   ** p < .01 *** p < .001 |  |  |  |  |  |

| Computational Modeling:  Punishment Learning Rate (Apun) |  |  |  |  |  |
| --- | --- | --- | --- | --- | --- |
|  |  | Model |  |  |  |
|  |  | 1 | 2 | 3 | 4 |
| **Fixed Effects** |  |  |  |  |  |
| Constant |  | -1.80 ^***^ [-1.83 – -1.77] | -1.81 ^***^ [-1.84 – -1.78] | -1.80 ^***^ [-1.84 – -1.76] | -1.81 ^***^ [-1.91 – -1.71] |
| Age |  | .02 [-.00 – .03] | .01 [-.02 – .03] | .01 [-.01 – .03] | .01 [-.01 – .03] |
| Age^2^ |  |  | .00 [-.00 – .01] |  |  |
| Maternal Hx Depression |  |  |  | -.00 [-.07 – .06] | -.02 [-.08 – .04] |
| Age * Maternal Hx Depression |  |  |  | .02 [-.01 – .06] | .02 [-.01 – .06] |
| K-BIT Nonverbal Standard IQ |  |  |  |  | .01 ^***^ [.00 – .01] |
| Child Sex |  |  |  |  | .01 [-.05 – .07] |
| On-site Administration |  |  |  |  | .01 [-.08 – .09] |
| **Random Effects** |  |  |  |  |  |
| σ^2^ |  | .06 | .06 | .06 | .06 |
| τ_00_ _id_ |  | .02 | .02 | .02 | .01 |
| τ_11_ _id.age_ |  | .00 | .00 | .00 | .00 |
| N _id_ |  | 208 | 208 | 208 | 208 |
| **Fit Statistics** |  |  |  |  |  |
| AIC |  | 248.2 | 248.4 | 250.6 | 226.9 |
| BIC |  | 270.7 | 275.5 | 282.2 | 272.0 |
| Loglik |  | -119.1 | -118.2 | -118.3 | -103.5 |
| Deviance |  | 238.2 | 236.4 | 236.6 | 206.9 |
| **Model Comparisons** |  |  |  |  |  |
| compared to Model 1 |  |  | $X_{1}^{2}$ = 1.75 | $X_{2}^{2}$ = 1.53 | $X_{5}^{2}$ = 31.23 ^***^ |
| compared to Model 2 |  |  |  |  |  |
| compared to Model 3 |  |  |  |  | $X_{3}^{2}$ = 29.70 ^***^ |
| ICC |  | .20 | .21 | .20 | .15 |
| Pseudo R^2^ (total) |  | .21 | .22 | .22 | .24 |
| * p < .05   ** p < .01 *** p < .001 |  |  |  |  |  |

| Computational Modeling:  Win Frequency Effect (BetaF) |  |  |  |  |  |
| --- | --- | --- | --- | --- | --- |
|  |  | Model |  |  |  |
|  |  | 1 | 2 | 3 | 4 |
| **Fixed Effects** |  |  |  |  |  |
| Constant |  | 1.71 ^***^ [1.36 – 2.06] | 1.64 ^***^ [1.29 – 2.00] | 1.82 ^***^ [1.38 – 2.25] | 4.42 ^***^ [3.25 – 5.59] |
| Age |  | .47 ^***^ [.26 – .68] | .31 ^*^ [.04 – .58] | .46 ^***^ [.20 – .72] | .31 ^*^ [.06 – .56] |
| Age^2^ |  |  | .08 [-.00 – .15] |  |  |
| Maternal Hx Depression |  |  |  | -.29 [-1.02 – .44] | -.52 [-1.21 – .17] |
| Age * Maternal Hx Depression |  |  |  | .01 [-.45 – .46] | .04 [-.39 – .47] |
| K-BIT Nonverbal Standard IQ |  |  |  |  | .05 ^***^ [.03 – .07] |
| Child Sex |  |  |  |  | -.10 [-.75 – .56] |
| On-site Administration |  |  |  |  | -2.50 ^***^ [-3.51 – -1.48] |
| **Random Effects** |  |  |  |  |  |
| σ^2^ |  | 8.26 | 8.18 | 8.27 | 8.15 |
| τ_00_ _id_ |  | 1.73 | 1.74 | 1.70 | 1.21 |
| τ_11_ _id.age_ |  | .92 | .94 | .92 | .75 |
| N _id_ |  | 208 | 208 | 208 | 208 |
| **Fit Statistics** |  |  |  |  |  |
| AIC |  | 3564.3 | 3562.8 | 3567.6 | 3531.3 |
| BIC |  | 3586.8 | 3589.9 | 3599.2 | 3576.4 |
| Loglik |  | -1777.1 | -1775.4 | -1776.8 | -1755.6 |
| Deviance |  | 3554.3 | 3550.8 | 3553.6 | 3511.3 |
| **Model Comparisons** |  |  |  |  |  |
| compared to Model 1 |  |  | $X_{1}^{2}$ = 3.41 | $X_{2}^{2}$ = .62 | $X_{5}^{2}$ = 42.99 ^***^ |
| compared to Model 2 |  |  |  |  |  |
| compared to Model 3 |  |  |  |  | $X_{3}^{2}$ = 42.36 ^***^ |
| ICC |  | .17 | .18 | .17 | .13 |
| Pseudo R^2^ (total) |  | .23 | .25 | .23 | .26 |
| * p < .05   ** p < .01 *** p < .001 |  |  |  |  |  |

| Computational Modeling:  Go Bias (BetaB) |  |  |  |  |  |
| --- | --- | --- | --- | --- | --- |
|  |  | Model |  |  |  |
|  |  | 1 | 2 | 3 | 4 |
| **Fixed Effects** |  |  |  |  |  |
| Constant |  | .86 ^***^ [.81 – .92] | .84 ^***^ [.79 – .90] | .87 ^***^ [.80 – .94] | 1.25 ^***^ [1.08 – 1.43] |
| Age |  | .09 ^***^ [.07 – .12] | .05 ^*^ [.01 – .08] | .05 ^*^ [.00 – .10] | .03 [-.01 – .08] |
| Age^2^ |  |  | .02 ^***^ [.01 – .03] | .02 ^**^ [.01 – .03] | .01 ^*^ [-.00 – .03] |
| Maternal Hx Depression |  |  |  | -.07 [-.18 – .05] | -.08 [-.20 – .03] |
| Age * Maternal Hx Depression |  |  |  | -.01 [-.09 – .07] | -.01 [-.09 – .07] |
| Age^2^ * Maternal Hx Depression |  |  |  | .00 [-.02 – .02] | .00 [-.02 – .03] |
| K-BIT Nonverbal Standard IQ |  |  |  |  | .00  [.00 – .01] |
| Child Sex |  |  |  |  | -.06 [-.16 – .04] |
| On-site Administration |  |  |  |  | -.33 ^***^ [-.48 – -.19] |
| **Random Effects** |  |  |  |  |  |
| σ^2^ |  | .17 | .16 | .16 | .17 |
| τ_00_ _id_ |  | .07 | .06 | .06 | .05 |
| τ_11_ _id.age_ |  | .01 | .01 | .01 | .01 |
| N _id_ |  | 208 | 208 | 208 | 208 |
| **Fit Statistics** |  |  |  |  |  |
| AIC |  | 954.4 | 944.1 | 948.5 | 933.3 |
| BIC |  | 977.0 | 971.2 | 989.1 | 987.4 |
| Loglik |  | -472.2 | -466.1 | -465.3 | -454.6 |
| Deviance |  | 944.4 | 932.1 | 930.5 | 909.3 |
| **Model Comparisons** |  |  |  |  |  |
| compared to Model 1 |  |  | $X_{1}^{2}$ = 12.26 ^***^ |  |  |
| compared to Model 2 |  |  |  | $X_{4}^{2}$ = 1.61 | $X_{6}^{2}$ = 22.88 ^***^ |
| compared to Model 3 |  |  |  |  | $X_{3}^{2}$ = 21.27 ^***^ |
| ICC |  | .29 | .28 | .27 | .22 |
| Pseudo R^2^ (total) |  | .37 | .40 | .39 | .35 |
| * p < .05   ** p < .01 *** p < .001 |  |  |  |  |  |
